# Parental intention to vaccinate daughters with the human papillomavirus vaccine in Korea: a nationwide cross-sectional survey

**DOI:** 10.4178/epih.e2023076

**Published:** 2023-08-17

**Authors:** Yejin Ha, Kyeongmin Lee, Bomi Park, Mina Suh, Jae Kwan Jun, Kui Son Choi

**Affiliations:** 1 Department of Cancer Control and Population Health, Graduate School of Cancer Science and Policy, National Cancer Center, Goyang, Korea; 2 Department of Preventive Medicine, Chung-Ang University College of Medicine, Seoul, Korea; 3 National Cancer Control Institute, National Cancer Center, Goyang, Korea

**Keywords:** Human papillomavirus, Vaccination, Intention

## Abstract

**OBJECTIVES:**

We aimed to identify and compare the characteristics and factors associated with parental intention to vaccinate daughters under 12 years old against human papillomavirus (HPV), examining data from 2016 and 2020.

**METHODS:**

Data were obtained from the Korean National Cancer Screening Survey conducted in 2016 and 2020. The present study included 3,510 parents with daughters under 12 years old. Changes in parental intention-to-vaccinate rates were calculated. To identify factors associated with parental intention to vaccinate their daughters, the chi-square test and logistic regression analysis were used.

**RESULTS:**

The percentage of respondents intending to vaccinate their daughters increased from 33.4% in 2016 to 58.9% in 2020, constituting a 25.5 percentage point (%p) increase. Since 2016, the proportion of men expressing positive intention towards HPV vaccination increased by 31.5%p, while that of women demonstrated a 20.9%p increase. Logistic regression analysis indicated that parents with a strong intention to vaccinate their daughters tended to be younger, more educated, and aware of the free vaccination program available, as well as to have a history of HPV vaccination and to have undergone cervical cancer screening within 2 years, compared to those who did not intend to vaccinate. Being a mother with a history of HPV vaccination was the strongest predictor of positive intention to vaccinate a daughter.

**CONCLUSIONS:**

The intention among parents to vaccinate daughters remains relatively low, although it is rising. To increase the HPV vaccination rate, strong recommendations and education should be provided to parents and the younger generation.

## GRAPHICAL ABSTRACT

[Fig f1-epih-45-e2023076]

## INTRODUCTION

Human papillomavirus (HPV) is among the most common sexually transmitted infections. Persistent infections with oncogenic or high-risk types of HPV can result in precancerous lesions and cancers, including those of the cervix, vulva, vagina, penis, anus, and oropharynx [1-3]. The majority of cervical cancer cases are caused by HPV infection, with HPV DNA found in approximately 95% of malignant cervical lesions [4]. In 2019, cervical cancer was the third most common cancer among women aged 15-34 years in Korea [5]. However, the overall incidence of cervical cancer has been on a downward trend. It decreased steadily until 2001 (annual percent change [APC], -1.0%), followed by a significant drop until 2007 (APC, -5.4%; p<0.05) and a slower decline through 2019 (APC, -2.8%; p<0.05) [5,6].

Fortunately, HPV vaccination can prevent cervical cancer [7]. Prophylactic HPV vaccines are highly effective in preventing most HPV infections that can lead to pre-invasive and invasive cervical cancer [8]. The World Health Organization (WHO) advocates for the inclusion of the HPV vaccine in National Immunization Programs (NIPs), with the primary target population for HPV vaccination being girls aged 9-14 years, prior to becoming sexually active [3]. In Korea, quadrivalent and bivalent HPV vaccines were approved in 2007 and 2008 [9], respectively, and HPV vaccination was incorporated into the NIP in June 2016 for 12-year-old girls, following a 2-dose schedule [10].

The Korea Disease Control and Prevention Agency recommends a 2-dose HPV regimen of the HPV vaccine. In 2016 and 2017, 61.5% and 72.7% of 12-year-old girls, respectively, received the first dose of the HPV vaccine series [11]. By 2019, this figure had risen to 83.5% [12], indicating an increasing trend in initiating the HPV series. Moreover, the percentage of 12-year-old girls completing the vaccination series increased from 54.7% for those born in 2003 to 79.8% for those born in 2007 [13]. However, when compared to the 95-98% completion rate for the infant vaccination series included in the NIP (excluding the Japanese encephalitis vaccine), the HPV vaccination rate remains low [14]. This discrepancy is often attributed to safety concerns as well as a lack of knowledge and awareness about HPV vaccination, factors that affect parental intent to initiate the HPV vaccine series [15].

Given that the national HPV vaccination program is aimed at 12-year-old girls, the decision to vaccinate is linked to their parents’ perceptions of and attitudes toward the HPV vaccine. Therefore, parental attitudes towards vaccination should be a primary focus for interventions promoting these vaccines.

A previous study was conducted to analyze and compare the awareness, attitudes, and parental intentions towards HPV vaccination in Korea in 2007 and 2016 [16]. However, since then, no studies have been conducted on changes in parental intentions. Therefore, our aim was to identify and compare the characteristics and factors associated with parental intention to vaccinate daughters under the age of 12 years in 2016, when HPV vaccination was incorporated into the Korean NIP, and in 2020.

## MATERIALS AND METHODS

### Data source and study population

Data were obtained from the Korean National Cancer Screening Survey (KNCSS) for the years 2016 and 2020. The KNCSS is a cross-sectional survey conducted annually by the Korean National Cancer Center. It targets cancer-free men and women aged 40-74 years and 20-74 years, respectively. The survey has been ongoing since 2004. Study participants are selected through a stratified multistage random sampling process. This process is based on resident registration population data, considering geographic area, age, and gender [17]. Participant recruitment was performed by a professional research agency via door-to-door contact. Face-to-face interviews were then conducted. At least 3 contact attempts were made per household, and 1 member was selected from each. The sampling methods have been detailed further in previous studies [17,18]. Among the KNCSS respondents, men and women aged 30-64 years with a daughter under the age of 12 years were included in the final analysis for the present study. The number of respondents was 4,500 in 2016 and 4,600 in 2020. Of these, 3,434 (76.3%) and 3,377 (73.4%) were 30-64 years old in 2016 and 2020, respectively. Among the respondents aged 30-64 years, the final analysis included 2,307 (67.2%) for 2016 and 1,203 (35.6%) for 2020. The number of participants included in the final analysis for 2020 was considerably smaller than for 2016, due to fewer respondents indicating that they had a daughter under the age of 12 years.

### Measures

All participants were asked to complete a structured questionnaire. This was designed to gather information on socio-demographic characteristics, intention toward HPV vaccination, and awareness of the NIP’s inclusion of HPV vaccination, which is provided free of charge.

Intention toward HPV vaccination was measured using a 5-point Likert-type scale. The question posed was “Would you be willing to vaccinate your daughter against the human papillomavirus when she reaches the age of 12 years?” Participants who chose “strongly agree” or “agree” were classified as intending to vaccinate their daughters. Conversely, those who responded with “strongly disagree,” “disagree,” or “neutral” were classified as not intending to vaccinate their daughters.

To measure awareness of complimentary HPV vaccination, we asked study participants, “Are you aware that the NIP offers free HPV vaccination for 12-year-old girls, given that the HPV vaccine is most effective before sexual activity begins?” Furthermore, we posed the following questions exclusively to women: “Have you ever received the HPV vaccine?”, “Have you ever undergone a cervical cancer screening using the Pap test?”, and “If so, when did you last undergo the Pap test?” Based on the responses to these questions, we could identify those who had undergone cervical cancer screening in accordance with the recommendations of the Korean National Cancer Screening Protocol within the prior 2 years.

### Statistical analysis

Descriptive statistics were employed to summarize participant characteristics and intention to vaccinate their daughters, with these presented as frequencies and percentages. The changes in the parental intention rate (expressed as percentage points [%p]) were calculated by subtracting the rate in 2020 from the rate in 2016 for all participants, as well as for men and women separately. The chi-square test was utilized to examine differences in parental intention rates between 2016 and 2020. Logistic regression was used to investigate the associations between parental intent to vaccinate their daughters and factors such as age, education level, monthly household income level, area of residence, and other vaccine-related and screening-related factors. Adjusted odds ratios (aORs) and 95% confidence intervals (CIs) were computed. All analyses were performed using SAS version 9.4 (SAS Institute Inc., Cary, NC, USA).

### Ethics statement

This study received approval from the Institutional Review Board of the National Cancer Center in Korea (approval No. NCC2019-0233). The participants consented to participate in the survey for public benefit, and the requirement for written informed consent was therefore waived.

## RESULTS

Table 1 presents the socio-demographic, behavioral, and perceptual characteristics of the study participants in 2016 and 2020. The distributions of gender and age were comparable in the 2016 and 2020 surveys. In the 2020 survey, participants demonstrated a significantly increased awareness of the free vaccination program compared to those in the 2016 survey. Furthermore, they reported higher rates of HPV vaccination and cervical cancer screening history.

Table 2 compares the parental intent to vaccinate daughters against HPV in 2016 and 2020. The proportion of respondents willing to vaccinate their daughters rose from 33.4% in 2016 to 58.9% in 2020, an increase of 25.5%p. Notably, the percentage of men with positive intention toward HPV vaccination increased by 31.5%p, from 28.6% in 2016 to 60.1% in 2020. In contrast, women exhibited a smaller increase of 20.9%p, from 37.1% in 2016 to 58.0% in 2020. Among men, those aged 50-64 years saw a larger increase in vaccination intent, from 22.0% to 57.3% (a 35.3%p increase), compared to those 40-49 years old (a 27.3%p increase). Among women, those aged 30-39 years experienced the largest increase in vaccination intent compared to other age groups, rising from 41.6% in 2016 to 69.4% in 2020 (a 27.8%p increase). In terms of education level, those who had completed university or graduate school demonstrated a greater increase in vaccination intent from 2016 to 2020 among all respondents, men, and women (with increases of 31.8, 36.5, and 28.2%p, respectively), compared to those with a lower level of education. The proportion of participants with a monthly household income exceeding US$4,500 who were willing to vaccinate their daughters saw a large increase, from 31.2% in 2016 to 61.0% in 2020.

Those who were aware of the national free HPV vaccination program were more likely to intend to vaccinate their daughters compared to those who were unaware. Moreover, the intention to vaccinate among those aware of the free HPV vaccination program rose from 43.9% in 2016 to 76.7% in 2020, an increase of 32.8%p. This increase was larger than that observed among those who were unaware of the program (21.0%p). Additionally, among women, those who had received the HPV vaccine were more likely to intend to vaccinate their daughters compared to those who had not been vaccinated. The intention to vaccinate among these women saw the greatest increase, from 48.8% in 2016 to 94.0% in 2020 (45.2%p). Furthermore, women who had undergone cervical cancer screening were more likely to intend to vaccinate their daughters compared to those who had not been screened. We noted a 22.1%p increase in vaccination intention among these women, from 40.3% in 2016 to 62.4% in 2020.

Table 3 presents the factors associated with parental intention to vaccinate their daughters, as per the 2020 survey. Men were 1.54 times (95% CI, 1.18 to 2.01) more likely to express an intention to vaccinate their daughters compared to women. Furthermore, respondents aged 30-39 years (aOR, 1.57; 95% CI, 1.02 to 2.40) were more likely to report intent to initiate the HPV vaccine series for their daughters than those aged 50-64 years. The results also indicated that respondents who had completed university or graduate school and those who were aware of the free HPV vaccination program were 1.37 (95% CI, 1.05 to 1.80) and 3.20 (95% CI, 2.37 to 4.30) times more likely, respectively, to plan to vaccinate their daughters compared to those who had completed only high school or a lower level of education and were unaware of the national program. Among the women, those who had received the HPV vaccine (aOR, 7.35; 95% CI, 2.57 to 21.04) and those who had undergone cervical cancer screening within the prior 2 years (aOR, 1.44; 95% CI, 1.03 to 2.03) were more likely to intend to vaccinate their daughters than participants who had not been vaccinated against HPV and those who had not undergone cervical cancer screening within that interval.

## DISCUSSION

The present study was conducted to compare parental intentions toward HPV vaccination in 2016, when HPV vaccination was incorporated into Korea’s NIP, and in 2020. Additionally, we analyzed factors associated with the intention to vaccinate daughters. Utilizing data from the KNCSS, we discovered an upward trend in the percentage of parents intending to vaccinate their daughters under 12 years old, increasing from 33.4% in 2016 to 58.9% in 2020. A previous study comparing parental intent to vaccinate against HPV in Korea found a decrease in parental willingness to vaccinate their daughters, from 77.0% in 2007 to 69.5% in 2016 [16]. These percentages are much higher than those obtained in the current study. The discrepancy in the 2016 results may be partially attributed to differences in the study questionnaires. Specifically, a previous study by Oh et al. [16] asked participants about the perceived benefits of HPV vaccination, based on the health belief model. Questions about perceived benefits included respondents’ willingness to vaccinate their daughters against HPV if the vaccine could prevent HPV infection. Moreover, Oh et al. [16] did not restrict their study participants to parents of 12-year-olds. In contrast, our study specifically asked parents of daughters under 12 years about their “actual intentions to vaccinate their daughters against HPV.” These differences limit the direct comparison and interpretation of the results of these studies.

Previous research conducted in the United States has indicated an improvement in parental intent and knowledge regarding vaccination over time. The National Immunization Survey-Teen (NIS-Teen) 2010-2015 revealed a significant decrease in the percentage of parents of unvaccinated girls who were “not at all likely” to vaccinate their teens, dropping from 41.5% to 31.2% (p<0.001). Conversely, it indicated an increase in those who were “somewhat likely” to vaccinate, rising from 16.3% to 22.0% (p<0.001) [19]. However, the actual vaccination rate remained low. According to the NIS-Teen survey, 37.1% of children aged 13-17 years remained unvaccinated. Alarmingly, among the parents of these unvaccinated adolescents, 58.0% had no intention of vaccinating their children [15]. The US Centers for Disease Control and Prevention (CDC) recommends that children aged 11 years to 12 years receive 2 doses of the HPV vaccine [20]. Furthermore, 5 jurisdictions have implemented HPV vaccination mandates for school attendance [21]. The CDC lists the cost of the HPV vaccine as US$208.5 per dose. However, most health insurance plans cover this cost, and those who are uninsured, underinsured, Medicaid-eligible, American Indian, or Alaska Native can access the HPV vaccine at a lower cost or for free through the Vaccines for Children program [20]. Despite the implementation of HPV vaccination systems in several states, a national HPV vaccination program that covers all children in the United States does not exist. This lack of a unified program contributes to the suboptimal HPV vaccination rate.

In Korea, the HPV vaccine was introduced into the NIP in 2016, and since then, it has been freely provided to girls aged 12 years and older [10]. Despite this, the completion rate for the vaccination series remains unsatisfactory. The proportion of individuals who received the initial dose of the HPV vaccine series rose from 61.5% in 2016 to 83.0% in 2022. However, only 60% to 70% of these individuals completed the entire HPV vaccine series [11,22, 23]. One potential reason for this situation could be the spread of misinformation about the HPV vaccine in the media, which has sparked concerns about its safety and potential side effects. For instance, in Japan, unverified reports of adverse events related to the vaccine and negative information in the media led to a sharp drop in the HPV vaccination rate [24,25]. This incident also caused hesitation among the unvaccinated population in Korea, deterring some from receiving the vaccine [26,27]. These safety concerns, fueled by misinformation, may result in a decrease in parental willingness to vaccinate their children, ultimately hindering an increase in the HPV vaccination rate.

The findings of this study highlight the factors linked to parental intention to vaccinate their daughters in 2020. We observed that parents with a strong intention to vaccinate their daughters were more likely to be younger, more educated, and aware of the free vaccination program, as well as to have a history of HPV vaccination and cervical cancer screening within the prior 2 years, relative to those who did not plan to vaccinate their daughters. These findings align with those of previous studies [16]. Interestingly, this study revealed that men were more likely to intend to vaccinate their children than women. In 2016, women demonstrated higher intention to vaccinate their daughters compared to men. However, by 2020, men reported a greater rate of intention to vaccinate. From 2016 to 2020, the proportion of men with a positive attitude towards HPV vaccination rose by 31.5%p, while women showed a comparatively smaller increase of 20.9%p. Regrettably, this study did not identify the reason for such a divergent rate of increase. Further research is required to explore this issue.

In comparing factors associated with parental intent to vaccinate against HPV in various countries, it was found that in the United States, parental knowledge of the HPV vaccine and maternal experience with Pap tests (for cervical cancer screening) were positively associated with intent to initiate and complete the HPV vaccine series [28]. In contrast to Korea, Norway, which has a coverage rate exceeding 90% for all childhood vaccines except the HPV vaccine, displayed positive associations between maternal income and knowledge of HPV with parental intent to vaccinate their daughters. Interestingly, a higher level of maternal education was negatively associated with parental intention to vaccinate [29]. The study found it challenging to explain this negative association between maternal education level and parental intent. Unlike in Norway, in Korea, maternal income was not identified as a key factor influencing parental vaccination intent. Across both Korea and other countries, parental knowledge of HPV vaccination and previous experience with cervical cancer screening were common factors that positively influenced parental intent to vaccinate their children.

The current study indicates that educating parents about HPV vaccination and cervical cancer prevention is important in increasing parental intent and the HPV vaccination rate. When parents consult doctors for screenings, they should be informed about the importance and effectiveness of HPV vaccination in preventing cervical cancer in their children. Moreover, our results suggest that awareness of the government’s free vaccination program was a significant factor influencing parental intention. This suggests that broadening the eligibility criteria for free HPV vaccination or actively advertising the availability of free HPV vaccines could significantly increase vaccination intention.

In March 2022, the government broadened the target demographic for the HPV vaccination from girls aged 12 years to include those aged 12-17 years, as well as girls aged 18-26 years from low-income households [22]. This expansion will undoubtedly enable more individuals to receive the HPV vaccine. However, the majority of people remain uninformed about this policy change due to a lack of broad promotion. In 2018, the WHO unveiled a global strategy for the elimination of cervical cancer. The strategy includes the following targets: 90% of girls should be fully vaccinated with the HPV vaccine by the age of 15 years, 70% of women should be screened using a high-performance test by the age of 35 years and again by the age of 45 years, 90% of women with pre-cancer should be treated, and 90% of women with invasive cancer should be managed. The WHO advises each country to achieve these 90-70-90 targets by 2030 in order to eradicate cervical cancer within the next century [30]. To align with the global objective of the WHO, the rate of HPV vaccination must be accelerated. Furthermore, it is crucial to educate parents about the prevention of cervical cancer, underscoring the pivotal role of the government in this endeavor.

This study sought to analyze and compare the intentions of parents to vaccinate their daughters, examining adults within the general population aged 30-64 years. We utilized nationwide data, employing stratified multistage random sampling based on resident registration population data. These data were categorized by geographic area, age, and gender, ensuring that the study’s data accurately represented the general population. Nevertheless, our study had several limitations. First, the data concerning demographic characteristics, as well as answers to survey questions about the history of HPV vaccination and cervical cancer screening, were self-reported. This could potentially introduce recall bias, as participants may not accurately remember their experiences or status. Second, the survey questions posed to parents with daughters in 2016 and 2020 were not identical. In 2016, parents were asked, “Are you willing to have your daughter vaccinated against the human papillomavirus through the NIP when she turns 12 years old in the future?” However, in 2020, the initial question was, “Do you have a daughter under the age of 12?” If the response was affirmative, the intention to vaccinate was then assessed. Consequently, in the 2016 survey, it is possible that parents with daughters over the age of 12 years or parents without daughters answered the question, while in the 2020 survey, only parents with daughters under 12 years old could respond. In fact, considering the distribution of participants in the final analysis, the percentage of respondents aged 50-64 years in 2016 was 40.1%, which was slightly higher than in 2020 (37.3%). This suggests the possibility that parents of children aged 12 years or older were included in 2016. Third, we analyzed intentions toward HPV vaccination, which may not necessarily translate into actual behavior. Based on our data, we were unable to determine how many participants actually vaccinated their daughters when they reached the age of 12 years. Therefore, additional research is needed to determine whether parental intent correlates with actual vaccination rates. Finally, the intention to vaccinate is influenced by the perceived susceptibility and severity of the disease. However, these factors were not considered in the current study. It was challenging to measure the likelihood of HPV infection (susceptibility) and the severity of the disease in girls under 12 years old. Incorporating a broader theoretical framework and investigating these factors could provide a more comprehensive understanding. Future research should examine these factors and discuss their relevance.

In conclusion, despite the increase in parental intent and vaccination rates since the inclusion of the HPV vaccine in the NIP, the proportion remains low relative to other government-provided vaccines. To elevate the HPV vaccination rate, it is crucial to understand parental intent to initiate and complete the HPV vaccination series. Furthermore, robust recommendations, promotions, and education targeting both parents and the younger generation are essential to ultimately reduce the burden of cervical cancer.

## Figures and Tables

**Figure f1-epih-45-e2023076:**
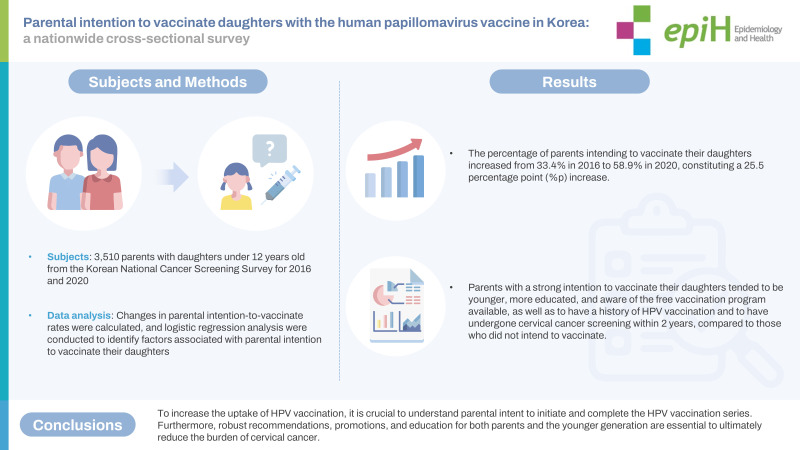


**Table 1.** Socio-demographic characteristics of the study participants (2016 and 2020)

Characteristics	Survey year		p-value
	2016	2020	
Total	2,307 (100)	1,203 (100)	
Gender			0.456
Men	991 (43.0)	501 (41.7)	
Women	1,316 (57.0)	702 (58.3)	
Age (yr)			0.014
30-39	423 (18.3)	193 (16.1)	
40-49	959 (41.6)	561 (46.6)	
50-64	925 (40.1)	449 (37.3)	
Education level			<0.001
High school or lower	1,327 (57.5)	600 (49.9)	
University or higher	980 (42.5)	603 (50.1)	
Monthly household income (USD)			<0.001
<2,990	457 (19.8)	115 (9.6)	
3,000-4,490	1,078 (46.7)	457 (38.0)	
≥4,500	772 (33.5)	631 (52.4)	
Area of residence			<0.001
Rural	355 (15.4)	46 (3.8)	
Urban	1,175 (50.9)	573 (47.6)	
Metropolitan	777 (33.7)	584 (48.6)	
Aware of free vaccination program			<0.001
No	1,742 (75.5)	834 (69.3)	
Yes	565 (24.5)	369 (30.7)	
History of HPV vaccination^1^			0.015
No	1,230 (93.5)	635 (90.5)	
Yes	86 (6.5)	67 (9.5)	
History of cervical cancer screening in line with recommendations^1,2^			0.004
No	581 (44.2)	263 (37.5)	
Yes	735 (55.9)	439 (62.5)	

Values are presented as number (%). USD, US dollar; HPV, human papillomavirus.

1 Applicable to women only.

2 Refers to cervical cancer screening for women aged ≥20 years within the previous 2 years, in accordance with Korean National Cancer Screening protocols.

**Table 2.** Participant intention to vaccinate their daughters by gender (2016 and 2020)

Variables		Overall		Change (%p)^1^	p-value^2^	Men		Change (%p)^1^	p-value^2^	Women		Change (%p)^1^	p-value^2^
		2016 (n=2,307)	2020 (n=1,203)			2016 (n=991)	2020 (n=501)			2016 (n=1,316)	2020 (n=702)		
Total		771 (33.4)	708 (58.9)	25.5	-	283 (28.6)	301 (60.1)	31.5	-	488 (37.1)	407 (58.0)	20.9	-
Gender													-
	Men	283 (28.6)	301 (60.1)	31.5	<0.001	-	-	-	-	-	-	-	
	Women	488 (37.1)	407 (58.0)	20.9	<0.001	-	-	-	-	-	-	-	
Age (yr)													
	30-39	176 (41.6)	134 (69.4)	27.8	<0.001	-	-	-	-	176 (41.6)	134 (69.4)	27.8	<0.001
	40-49	367 (38.3)	342 (61.0)	22.7	<0.001	176 (34.9)	179 (62.2)	27.3	<0.001	191 (42.1)	163 (59.7)	17.6	<0.001
	50-64	228 (24.6)	232 (51.7)	27.1	<0.001	107 (22.0)	122 (57.3)	35.3	<0.001	92 (27.1)	84 (48.3)	21.2	<0.001
Education level													
	High school or lower	441 (33.2)	313 (52.2)	19.0	<0.001	156 (27.9)	125 (53.4)	25.5	<0.001	285 (37.1)	188 (51.4)	14.3	<0.001
	University or higher	330 (33.7)	395 (65.5)	31.8	<0.001	127 (29.4)	176 (65.9)	36.5	<0.001	203 (37.0)	219 (65.2)	28.2	<0.001
Monthly household income (US dollar)													
	<2,990	123 (26.9)	57 (49.6)	22.7	<0.001	49 (24.1)	21 (52.5)	28.4	<0.001	74 (29.1)	36 (48.0)	18.9	0.002
	3,000-4490	407 (37.8)	266 (58.2)	20.4	<0.001	141 (31.5)	112 (56.6)	25.1	<0.001	266 (42.2)	154 (59.5)	17.3	<0.001
	≥4,500	241 (31.2)	385 (61.0)	29.8	<0.001	93 (27.4)	168 (63.9)	36.5	<0.001	148 (34.3)	217 (59.0)	24.7	<0.001
Area of residence													
	Rural	113 (31.8)	25 (54.4)	22.6	0.003	49 (27.5)	6 (40.0)	12.5	0.305	64 (36.2)	19 (61.3)	25.1	0.008
	Urban	307 (26.1)	306 (53.4)	27.3	<0.001	113 (22.5)	131 (54.1)	31.6	<0.001	194 (28.8)	175 (52.9)	24.1	<0.001
	Metropolitan	351 (45.2)	377 (64.5)	19.3	<0.001	121 (38.9)	164 (67.2)	28.3	<0.001	230 (49.4)	213 (62.7)	13.3	<0.001
Aware of free vaccination program													
	No	523 (30.0)	425 (51.0)	21.0	<0.001	235 (29.6)	233 (56.1)	26.5	<0.001	288 (30.4)	192 (45.8)	15.4	<0.001
	Yes	248 (43.9)	283 (76.7)	32.8	<0.001	48 (24.2)	68 (79.1)	54.9	<0.001	200 (54.5)	215 (76.0)	21.5	<0.001
History of HPV vaccination^3^													
	No	-	-	-	-	-	-	-	-	446 (36.3)	344 (54.2)	17.9	<0.001
	Yes	-	-	-	-	-	-	-	-	42 (48.8)	63 (94.0)	45.2	<0.001
History of cervical cancer screening in line with recommendations^3,4^													
	No	-	-	-	-	-	-	-	-	192 (33.0)	133 (50.6)	17.6	<0.001
	Yes	-	-	-	-	-	-	-	-	296 (40.3)	274 (62.4)	22.1	<0.001

Values are presented as number (%); Row percentage for an intention of “yes,” referring to participants who either strongly agreed or agreed to vaccinate their daughters.

1 Percentage change in the proportion of parents with strong intention to vaccinate from 2016 to 2020 (percentage point; %p).

2 p-value of the comparison of strong intention rate between groups, as determined using the chi-square test.

3 Applicable to women only.

4 Refers to cervical cancer screening for women aged ≥20 years within the previous 2 years, in accordance with Korean National Cancer Screening protocols.

**Table 3.** Factors associated with high parental intention to vaccinate compared to low intention (2020)

Variables		Overall^1^	Men^2^	Women^3^
Gender				
	Men	1.54 (1.18, 2.01)	-	-
	Women	1.00 (reference)	-	-
Age group (yr)				
	30-39	1.57 (1.02, 2.40)	-	1.90 (1.18, 3.06)
	40-49	1.14 (0.86, 1.50)	0.99 (0.66, 1.50)	1.40 (0.94, 2.08)
	50-64	1.00 (reference)	1.00 (reference)	1.00 (reference)
Education level				
	High school or lower	1.00 (reference)	1.00 (reference)	1.00 (reference)
	University or higher	1.37 (1.05, 1.80)	1.53 (1.01, 2.32)	1.24 (0.86, 1.79)
Monthly household income (USD)				
	<2,990	1.00 (reference)	1.00 (reference)	1.00 (reference)
	3,000-4,490	0.97 (0.63, 1.50)	0.84 (0.41, 1.72)	1.07 (0.60, 1.92)
	≥4,500	1.00 (0.64, 1.54)	1.07 (0.52, 2.20)	0.89 (0.50, 1.60)
Area of residence				
	Rural	1.00 (reference)	1.00 (reference)	1.00 (reference)
	Urban	1.37 (0.73, 2.58)	2.35 (0.79, 6.94)	0.95 (0.42, 2.14)
	Metropolitan	0.84 (0.45, 1.58)	1.34 (0.45, 3.95)	0.61 (0.27, 1.38)
Aware of free vaccination program				
	No	1.00 (reference)	1.00 (reference)	1.00 (reference)
	Yes	3.20 (2.37, 4.30)	2.82 (1.59, 4.99)	2.49 (1.73, 3.60)
History of HPV vaccination^4^				
	No	-	-	1.00 (reference)
	Yes	-	-	7.35 (2.57, 21.04)
History of cervical cancer screening in line with recommendations^4,5^				
	No	-	-	1.00 (reference)
	Yes	-	-	1.44 (1.03, 2.03)

Values are presented as adjusted odds ratio (95% confidence interval). HPV, human papillomavirus.

1 Adjusted for age, education level, monthly household income, area of residence, awareness of free vaccination program, history of HPV vaccination, and history of cervical cancer screening in line with recommendations for both men and women.

2 Adjusted for age, education level, monthly household income, area of residence, and awareness of free vaccination program for men.

3 Adjusted for age, education level, monthly household income, area of residence, awareness of free vaccination program, history of HPV vaccination, and history of cervical cancer screening in line with recommendations for women.

4 Applicable to women only.

5 Refers to cervical cancer screening for women aged ≥20 years within the previous 2 years, in accordance with Korean National Cancer Screening protocols.
